# Neuroepithelial cell competition triggers loss of cellular juvenescence

**DOI:** 10.1038/s41598-020-74874-4

**Published:** 2020-10-22

**Authors:** Faidruz Azura Jam, Takao Morimune, Atsushi Tsukamura, Ayami Tano, Yuya Tanaka, Yasuhiro Mori, Takefumi Yamamoto, Masaki Nishimura, Ikuo Tooyama, Masaki Mori

**Affiliations:** 1 Molecular Neuroscience Research Center (MNRC), Shiga University of Medical Science, Seta Tsukinowa-Cho, Otsu, Shiga 520-2192 Japan; 2 Department of Pediatrics, Shiga University of Medical Science, Seta Tsukinowa-Cho, Otsu, Shiga 520-2192 Japan; 3 Central Research Laboratory, Shiga University of Medical Science, Seta Tsukinowa-Cho, Otsu, Shiga 520-2192 Japan

**Keywords:** Cell biology, Cell proliferation

## Abstract

Cell competition is a cell–cell interaction mechanism which maintains tissue homeostasis through selective elimination of unfit cells. During early brain development, cells are eliminated through apoptosis. How cells are selected to undergo elimination remains unclear. Here we aimed to identify a role for cell competition in the elimination of suboptimal cells using an in vitro neuroepithelial model. Cell competition was observed when neural progenitor HypoE-N1 cells expressing RAS^V12^ were surrounded by normal cells in the co-culture. The elimination through apoptosis was observed by cellular changes of RAS^V12^ cells with rounding/fragmented morphology, by SYTOX blue-positivity, and by expression of apoptotic markers active caspase-3 and cleaved PARP. In this model, expression of juvenility-associated genes Srsf7 and Ezh2 were suppressed under cell-competitive conditions. *Srsf7* depletion led to loss of cellular juvenescence characterized by suppression of *Ezh2*, cell growth impairment and enhancement of senescence-associated proteins. The cell bodies of eliminated cells were engulfed by the surrounding cells through phagocytosis. Our data indicates that neuroepithelial cell competition may have an important role for maintaining homeostasis in the neuroepithelium by eliminating suboptimal cells through loss of cellular juvenescence.

## Introduction

One of the fundamental questions in developmental biology is whether there are mechanisms that can detect aberrant, defective, or mutant cells that compromise their capability to contribute to development. Occurrence of endogenous apoptosis is increased in early development, around embryonic day E6.5^1^, and this implies cell competition might contribute to selection of fitter cells and the elimination of unwanted cells. Cell competition has been reported in mouse embryos^2^ during epiblast formation^3^, in various organs such as epidermis^4^, heart^5^ and pancreas^3^, but is less understood in the nervous system.

Programmed cell death (PCD) has a significant functional role in nervous system development with apoptosis being the major form of PCD^6^. Senescence-undergoing cells are present in early developing embryos in mice, human^7^ and *Xenopus*^8^. As defective, senescent and unwanted cells need to be eliminated, a quality control mechanism to ensure proper tissue growth and homeostasis must exist. However, the molecular mechanisms underlying this process are unknown.

Cell competition is a conserved cell-eliminating mechanism for tissue homeostasis maintenance^9^, quality control^10^ and as a tumor suppressive mechanism^11^. The phenomenon was discovered more than 40 years ago in the wing disc of *Drosophila melanogaster*, in which *Minutes* mutants that exhibit defective protein synthesis were eliminated actively during development and disappeared from the adult body. This mechanism is evolutionarily conserved^12,13^.

Determination of cell fate between winner or loser is a non-cell autonomous property of a cell, and it is elicited by cellular interactions with neighbouring cells^14^. Cell fitness is an unquantifiable concept referring to quality of a cell, for example growth rate, differential activation of cell damage pathways and metabolic activity, although recent works have identified the relevance of several fitness markers such as for *flower*^15^ or *Sparc*^16^. Cell competition usually requires contact or close proximity^14^, as in *Minute-*mediated cell competition^17^, to provoke fitness comparison. Among important hallmarks of cell competition are context dependency, which are triggered by a local difference in cellular growth or metabolism, proliferation of winner cells stimulated by loss of loser cells, and short-range effects which are stronger at the interface between loser and winner cells^18^. Mechanisms for the loser-cell elimination are through apoptosis^19^, engulfment^17^, apical extrusion^20^ and basal extrusion^21^.

Well-characterized inducers of cell competition include *minute*^22^, *Bone morphogenetic proteins (BMPs)*^1^, *Myc*^2^, and *Ras*^20,23^. The discovery of *HRAS* (Harvey rat sarcoma virus) in 1964 was isolated from the passage of Moloney type-C virus in rats^24^*.* High expression of activated *HRAS oncogene* has been proposed to induce senescence^25^. An oncogene-induced senescence (OIS) has been reported by induction of an oncogenic form of *RAS* in normal human fibroblasts^26^.

Cellular juvenescence is characterized by the ability of cells to grow, differentiate and resist premature senescence^27^. We previously identified juvenility-associated genes (JAGs) expressed in brain^28^, cardiomyocytes and hepatocytes^29^. JAGs are genes selectively highly expressed in juvenile cells. *Srsf7*, Serine-Arginine-rich family of pre-mRNA splicing factors, which constitutes a part of the spliceosome, is one of the JAGs^29^.

In this communication, to investigate the role of cell competition in the developing brain, we have established an in vitro model system. In mice, formation of a neural plate begins at E7.5. Neural plate is an earliest indication of neural development. At this stage, cephalization begins with the presence of enlarged head folds and formation of neural plate from the ectoderm, which converts into neuroectoderm. Based on this developmental stage, we have established a heterogeneous neuroepithelial model in which neural progenitor HypoE-N1 cells were cultured as a monolayer. Heterogeneity was introduced by induction of RAS^V12^ in a small proportion of the cells. We investigated the molecular mechanisms in neuroepithelial cell competition by which juvenescence-losing cells are sensed and eliminated from the neuroepithelium. In this study, we clarify a role of cell competition as a quality-control mechanism to orchestrate homeostasis in the brain.

## Results

### Establishment of the neuroepithelial cell competition model

**Figure 1:**
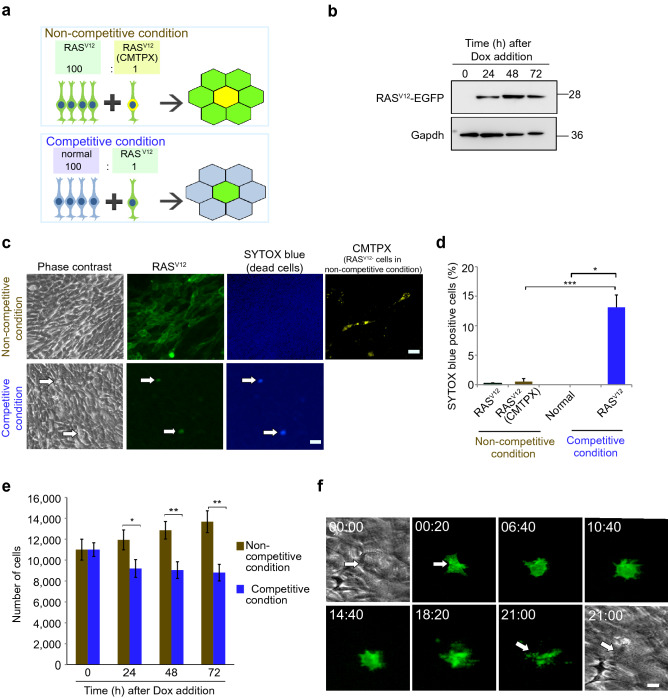
Establishment of the neuroepithelial cell competition model. (**a**) Experimental scheme for neuroepithelial cell competition. In non-competitive control condition, EGFP-expressing RAS^V12^ cells were co-cultured with RAS^V12^ labeled with CMTPX. In competitive condition, normal (wild type) cells were co-cultured with EGFP-RAS^V12^. The ratio of co-cultured cells for both conditions were 100:1. (**b**) Western blot analysis showing RAS^V12^ expression upon doxycycline induction. Gapdh was used as a loading control. (**c**) Representative images of SYTOX blue staining in non-competitive and competitive condition. Arrows indicate cells positive for SYTOX blue. Scale bars = 50 µm. (**d**) Frequency of SYTOX blue-positive cells in non-competitive and competitive condition. (**e**) Number of RAS^V12^ (CMTPX) in non-competitive and RAS^V12^ (EGFP) cells in competitive condition at 0, 24, 48 and 72 h. (**f**) Snapshot images of time-lapse movie showing elimination of RAS^V12^ cell surrounded by normal cells through cell death induction. Scale bar = 20 µm. Data are shown as mean ± SEM from three independent experiments. (Student’s t-test *p < 0.05, **p < 0.01, ***p < 0.001).

To investigate the phenomenon of cell competition in the developing mammalian central nervous system, we established a cellular model for neuroepithelium using HypoE-N1 cells, a neural progenitor cell line derived from mouse fetal hypothalamus. We performed conditioned medium assays and indirect co-culture assays to exclude the possibility of indirect effects. We confirmed that neuroepithelial cell competition requires direct cell–cell contact, and was not mediated by soluble factors produced during competitive co-culture (Supplementary Fig. S1). In this system, we co-cultured normal HypoE-N1 cells with doxycycline-inducible RAS^V12^-EGFP-transfected HypoE-N1 cells (hereinafter referred to as RAS^V12^ cells) at a 100:1 ratio to create competitive conditions. For the non-competitive condition, we co-cultured RAS^V12^ cells with RAS^V12^ cells labeled with the fluorescent dye CMTPX (Fig. 1a). The expression of RAS^V12^ upon doxycycline treatment was validated by western blot analysis. RAS^V12^ was expressed stably up to 72 h with doxycycline treatment (Fig. 1b). SYTOX blue staining showed RAS^V12^ cells in a competitive condition are positive, indicating they are dying (Fig. 1c). The percentage of SYTOX blue positive in RAS^V12^ cells in competitive condition were significantly higher compared to normal cells. However, in the non-competitive control condition, both RAS^V12^ and RAS^V12^ (CMTPX) cells showed lower frequency of SYTOX blue staining with no significant difference between them (Fig. 1d).

Next, we evaluated the effects of co-culture on the growth rate of cells. The changes in RAS^V12^ cell numbers over time were compared between competitive and non-competitive co-cultures. We found competitive co-cultures showed reduction in RAS^V12^ cell numbers after 24 h compared to RAS^V12^ (CMTPX) in the non-competitive condition (Fig. 1e). We then recorded the phenomenon occurring in the competitive co-culture. Time-lapse video revealed that RAS^V12^ cells surrounded by normal HypoE-N1 cells showed morphological changes involving rounding-up and eventually fragmentation (Fig. 1f, Supplementary Video S1). Collectively, our data suggest introduction of a small number of RAS^V12^ cells induced heterogeneity to the neuroepithelium and this established a model for cell competition with the loser RAS^V12^ cells being eliminated through non-cell autonomous cell death.

### Neuroepithelial cell competition occurs by non-cell autonomous induction of apoptosis

**Figure 2:**
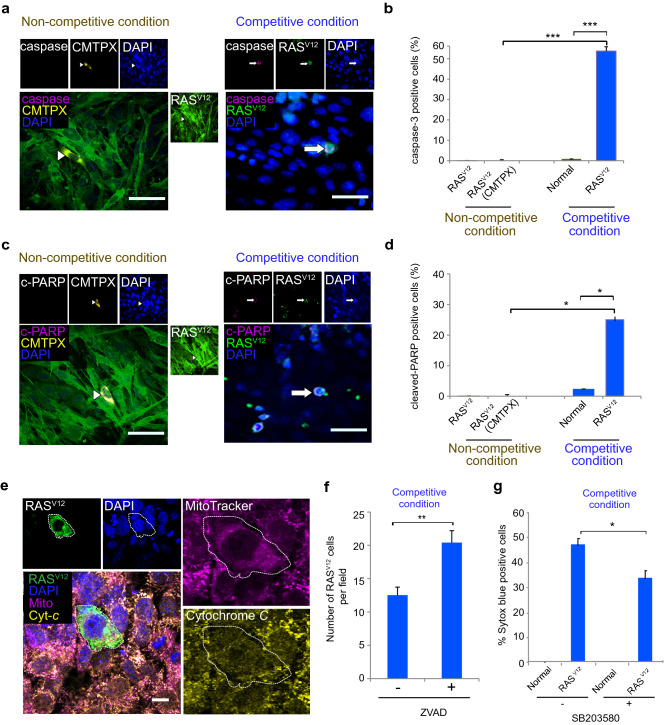
Neuroepithelial cell competition occurs by non-cell autonomous induction of apoptosis. (**a**) Representative images of caspase-3 in RAS^V12^ cells in non-competitive and competitive condition. Arrows indicate cells positive for caspase-3. Scale bars = 50 µm. (**b**) Frequency of caspase-3 positive cells in non-competitive and competitive condition. (**c**) Representative image of cleaved PARP in RAS^V12^ cells in non-competitive and competitive condition. Arrows indicate positive for cleaved PARP. Scale bars = 50 µm. (**d**) Frequency of cleaved PARP positive cells in non-competitive and competitive condition. (**e**) Cytochrome-*c* and Mitotracker CMXROS staining in competitive condition showed cytochrome-*c* was diffusely stained in addition to co-localization with mitochondria in RAS^V12^ cell surrounded by normal cells. Scale bar = 50 µm. (**f**) Number of RAS^V12^ cells per field in competitive condition in the absence and presence of apoptosis inhibitor ZVAD. (**g**) Frequency of cell death indicated by SYTOX blue positivity in the competitive condition in the absence and presence of p38 inhibitor SB203580. Data are shown as mean ± SEM from three independent experiments (Student’s t-test *p < 0.05, **p < 0.01, ***p < 0.001).

We next investigated the mechanism of RAS^V12^ cell death from competitive co-culture. During cell competition, most of the loser cells will be eliminated by apoptosis^30^. Thus, we asked whether RAS^V12^ cells in competitive co-culture were also eliminated by apoptosis. Immunostaining with apoptosis markers active caspase-3 and cleaved PARP displayed positive signals in RAS^V12^ cells surrounded by normal cells in the competitive co-cultures. By contrast, RAS^V12^ (CMTPX) cells in non-competitive co-cultures did not show positive signals for either apoptosis marker (Fig. 2a,c). Quantification for active caspase-3 and cleaved PARP-positive signals showed RAS^V12^ cells were significantly higher compared to normal cells in the competitive condition (Fig. 2b,d).

During apoptosis, cytochrome-*c* is released from mitochondria^31^. Healthy cells displayed punctate patterns of cytochrome-*c* staining that co-localized with mitochondria. In contrast, cells undergoing apoptosis showed a diffuse pattern of cytochrome-*c*. RAS^V12^ cells surrounded by normal cells in competitive co-culture displayed diffuse cytochrome-*c* staining that did not co-localize with mitochondria (Fig. 2e). We next tried to block apoptosis by using the apoptosis inhibitor ZVAD in the co-cultures. ZVAD treatment significantly increased the number of RAS^V12^ cells in the competitive co-culture compared to those without ZVAD treatment (Fig. 2f).

A previous study reported involvement of the p38 MAPK pathway during cell competition^32^. Immunofluorescence staining for phospho-p38 in the competitive condition indicated p38 was activated in RAS^V12^ cells (Supplementary Fig. S2). To answer whether apoptosis was induced through the MAPK pathway, we inhibited p38 activation in the competitive co-cultures. The percentage of SYTOX blue positive cells was significantly lower in the presence of p38 inhibitor (Fig. 2g). These results indicated that RAS^V12^ cells in competitive co-culture underwent cell death through non-cell autonomous apoptosis.

### Neuroepithelial cell competition triggers Srsf7 loss in RAS^V12^ cells via proteasome mediated degradation

**Figure 3:**
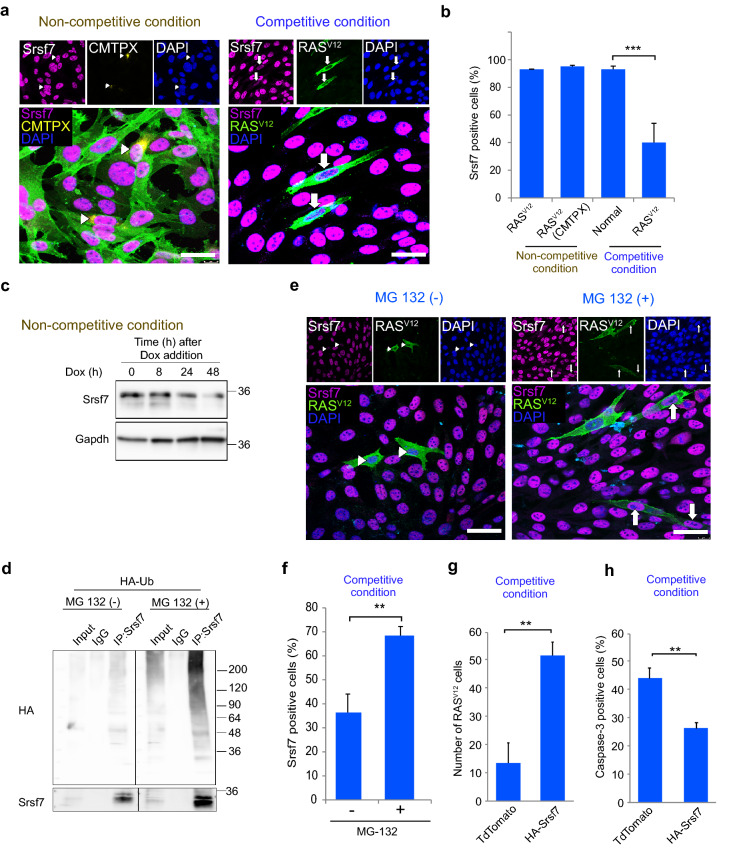
Neuroepithelial cell competition triggers Srsf7 loss in RAS^V12^ cells via proteasome-mediated degradation. (**a**) Representative images of Srsf7 expression in non-competitive and competitive condition. Arrows indicate Srsf7 loss in cells. Scale bars = 10 µm. (**b**) Frequency of Srsf7 positive cells in non-competitive and competitive condition. (**c**) Western blot analysis of Srsf7 expression in the non-competitive condition upon doxycycline induction. Gapdh was used as a loading control. (**d**) Ubiquitylation assay of Srsf7. Cells were transfected with HA-tagged ubiquitin and treated with MG132, 5 µM for 8 h before lysate collection. The ubiquitylation of Srsf7 was determined by IP-western. Note that samples were derived from the same experiment and blots were processed in parallel. Full length blots are presented in Supplementary Fig. S4. (**e**) Representative images of Srsf7 expression in the absence and presence of proteasome inhibitor MG132 during competitive condition. Arrows indicate Srsf7 positive cells. Scale bars = 20 µm. (**f**) Frequency of Srsf7 positive cells in the absence and presence of proteasome inhibitor MG132 during competitive condition. (**g**) Number of RAS^V12^ cells counted after 24 h in the competitive condition of normal cells with control TdTomato RAS^V12^ cells or HA-Srsf7 overexpressed RAS^V12^ cells. (**h**) Frequency of caspase-3 positive in control TdTomato RAS^V12^ cells or HA-Srsf7 overexpressed RAS^V12^ cells during competitive condition. Data are shown as mean ± SEM from three independent experiments (Student’s t-test **p < 0.01, ***p < 0.001).

We next explored the mechanisms underlying the elimination of RAS^V12^ from the competitive condition. First, we investigated protein expression of JAGs in cells by immunofluorescence analysis. We observed as early as 16 h of competitive condition, Srsf7 expression was lost in RAS^V12^ cells surrounded by normal cells (Fig. 3a). In the non-competitive condition, the percentage of Srsf7 positivity among RAS^V12^ and RAS^V12^ (CMTPX) cells was not significantly different, but in competitive co-cultures, the percentage of Srsf7 positive cells was significantly decreased in RAS^V12^ cells compared to normal cells (Fig. 3b). Western blot analysis of non-competitive condition cells showed Srsf7 expression was slightly reduced with RAS^V12^ induction (Fig. 3c). These results suggest competitive co-culture caused acute loss of Srsf7 in RAS^V12^ cells. We next explored how Srsf7 loss was faster in RAS^V12^ cells during the competitive condition. SR proteins have been reported to undergo post-translational modifications by phosphorylation, acetylation, methylation, ubiquitylation and sumoylation^33^. A previous study reported that SRSF5, one of the SR family members, was ubiquitylated by Smurf1 and undergoes proteasome-mediated degradation^34^. To assess whether Srsf7 is degraded through the ubiquitin-proteasome pathway, we conducted ubiquitylation assays. We found that the ubiquitin immunoreactive smear was significantly enhanced with MG132 treatment in HA-Ub transfected cells (Fig. 3d). To further analyze the loss of Srsf7 by proteasome-mediated degradation in the competitive condition, we treated the co-cultures with MG132 and quantified the number of Srsf7 positive in RAS^V12^ cells in the absence and presence of MG132 (Fig. 3e). The percentage of Srsf7 positive in RAS^V12^ cells with MG132 treatment was higher compared to the non-treated group (Fig. 3f). These observations indicated that during competitive co-culture, Srsf7 in RAS^V12^ cells was degraded through the ubiquitin-proteasome pathway. To investigate if restoring Srsf7 expression is sufficient to prevent RAS^V12^ cell elimination, we overexpressed Srsf7 in RAS^V12^ cells and co-cultured them with normal cells. Srsf7 overexpression in RAS^V12^ cells (HA-Srsf7) significantly increased the number of RAS^V12^ cells compared to control treatment (TdTomato, Fig. 3g). In addition, Srsf7 overexpression in RAS^V12^ cells (HA-Srsf7) significantly reduced the frequency of caspase-3 positive cells (Fig. 3h), which verifies that Srsf7 antagonizes the execution of cell competition. Collectively, these results revealed cell competition triggers the acute loss of Srsf7 in RAS^V12^ cells through proteasome-mediated degradation.

### Loss of Srsf7 suppresses Ezh2 expression in the loser cells

**Figure 4:**
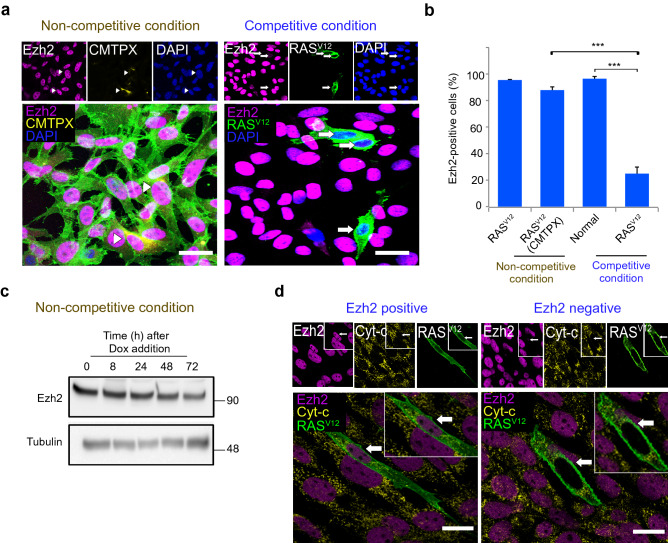
Loss of Srsf7 induces Ezh2 suppression in the loser cells. (**a**) Representative images of Ezh2 expression in non-competitive and competitive condition. Arrows indicate Ezh2 loss in cells. Scale bars = 20 µm. (**b**) Frequency of Ezh2 positive cells in non-competitive and competitive condition. (**c**) Western blot analysis of Ezh2 expression in the non-competitive condition upon doxycycline induction. Tubulin was used as a loading control. (**d**) Cytochrome-*c* staining showed punctate pattern in both RAS^V12^ cells with Ezh2 positive and Ezh2 negative cell. Scale bars = 20 µm. Data are shown as mean ± SEM from three independent experiments (Student’s t-test ***p < 0.001).

To investigate how the expression of other proteins change, we measured other JAGs protein expression after 24 h of co-culture. Competitive co-cultures displayed acute loss of Ezh2 in RAS^V12^ cells (Fig. 4a). The percentage of Ezh2-expressing RAS^V12^ cells in competitive condition was significantly lower compared to normal cells (Fig. 4b). This observation showed a similar pattern with Srsf7 expression in non-competitive and competitive condition. Western blot analysis revealed Ezh2 expression in non-competitive condition was slightly downregulated with RAS^V12^ induction up to 72 h (Fig. 4c). Next, we did immunofluorescence staining analysis for cytochrome-*c* to investigate whether Ezh2 loser cells underwent apoptosis. We found that both Ezh2 positive and negative RAS^V12^ cells showed punctate staining, which suggests that Ezh2 loss occurs before apoptosis induction (Fig. 4d). Thus, our analyses revealed Srsf7 loss induced Ezh2 suppression in loser cells and was a primary mechanism in neuroepithelial cell competition.

### Srsf7 depletion induces loss of cellular juvenescence through Ezh2

**Figure 5:**
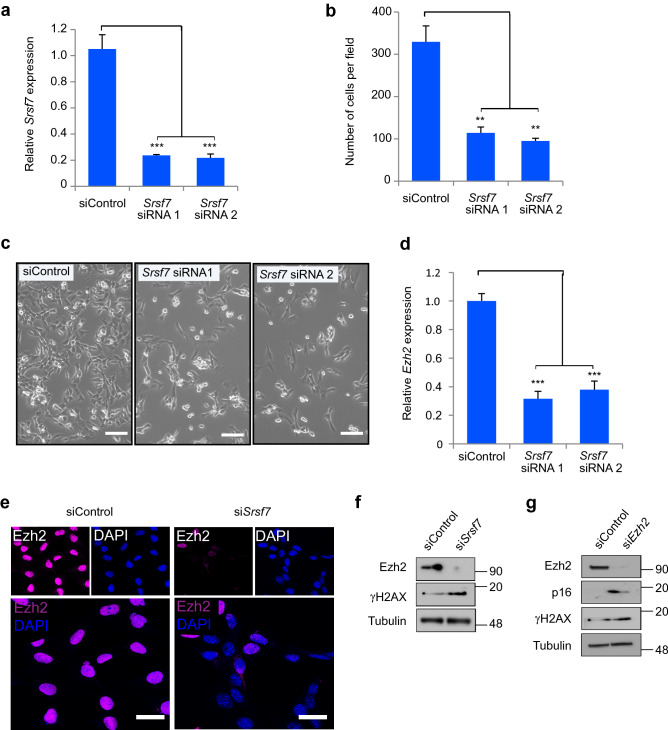
Srsf7 suppression induces loss of cellular juvenescence. (**a**) qPCR analysis of *Srsf7* knockdown efficiency in HypoE-N1 cells after 48 h transfection of control siRNA, *Srsf7* siRNA1 or siRNA2. (**b**) Number of cells per field 72 h after transfection with control siRNA or *Srsf7* siRNA1 and siRNA2. (**c**) Appearance of HypoE-N1 cells after transfection with control siRNA or *Srsf7* siRNA 1 and siRNA 2. Scale bars = 50 µm. (**d**) qPCR analysis of *Ezh2* mRNA expression 72 h after transfection with control siRNA, *Srsf7* siRNA1 or siRNA2. (**e**) Immunofluorescent staining of Ezh2 in HypoE-N1 cells 48 h after transfection with control siRNA or *Srsf7* siRNA. Scale bars = 20 µm. (**f**) Western blot analysis with HypoE-N1 cells 72 h after transfection with control siRNA or *Srsf7* siRNA. Tubulin was used a loading control. (**g**) Western blot analysis with HypoE-N1 cells 72 h after transfection with control siRNA or *Ezh2* siRNA. Tubulin was used a loading control. Data are shown as mean ± SEM from three independent experiments (Student’s t-test **p < 0.01, ***p < 0.001).

To elucidate the role of Srsf7 in cell competition, we depleted *Srsf7* in HypoE-N1 cells using gene-specific siRNA transfection. Knockdown efficiency was validated with quantitative PCR analysis (Fig. 5a). *Srsf7* depletion significantly impaired cell growth (Fig. 5b,c). We had previously identified *Srsf7* and *Ezh2* as JAGs^27^. *Ezh2* mRNA expression was downregulated upon *Srsf7* knockdown (Fig. 5d), which suggests Srsf7 regulates *Ezh2* transcription. Immunostaining showed Ezh2 expression was markedly reduced in *Srsf7*-depleted cells (Fig. 5e). This finding was corroborated by western blot analysis which showed Ezh2 (one of the JAGs) protein levels were suppressed in the *Srsf7*-depleted cells, while the expression of gamma H2AX (senescence-associated protein) was increased (Fig. 5f). To analyze how cellular juvenescence was regulated by *Ezh2*, we tested the consequences of *Ezh2* depletion on HypoE-N1 cells. siRNA-mediated knockdown of *Ezh2* led to increased expression of senescence-associated proteins P16 and gamma H2AX (Fig. 5g). To further test whether Srsf7 suppression was sufficient to induce cell competition, we co-cultured *Srsf7*-knocked down in normal HypoE-N1 cells with normal HypoE-N1 cells. We found the number of *Srsf7*-knocked down cells was reduced compared to control siRNA transfected cells. Furthermore, we observed a significant increase of caspase-3 positive in *Srsf7*-knocked down cells (Supplementary Fig. S3). These findings indicate *Srsf7* depletion in loser RAS^V12^ cells induces loss of cellular juvenescence through *Ezh2* suppression and is sufficient to induce cell competition.

### Neuroepithelial cell competition removes loser cell corpora via phagocytosis

**Figure 6:**
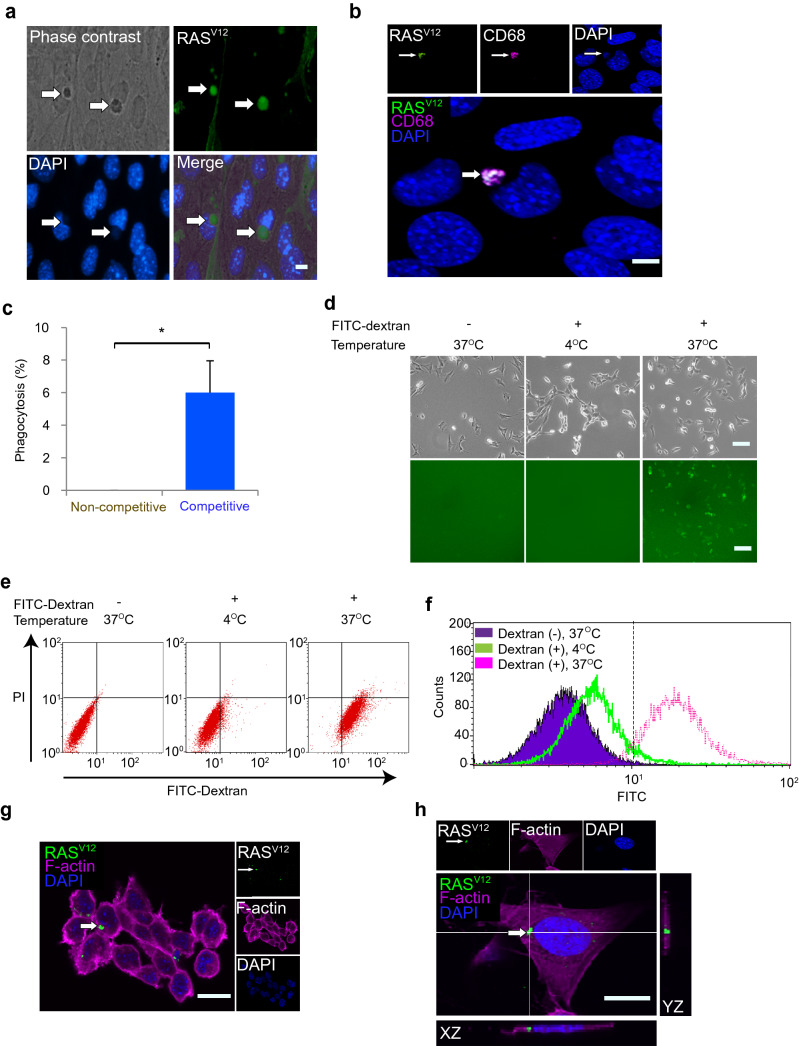
Neuroepithelial cell competition removes loser cell corpora via phagocytosis. (**a**) Representative image of RAS^V12^ cell corpora phagocytized by surrounding normal HypoE-N1 cell. Arrows indicate RAS^V12^ cells corpora. Scale bar = 10 µm. (**b**) Representative image of co-localization RAS^V12^ cell corpora with CD68 (phagosome marker), depicted by arrows. Scale bar = 5 µm. (**c**) Percentage of phagocytosis at 48 h, counted from co-localization of CD68 and RAS^V12^ cell corpora. (**d**) FITC-dextran uptake assay. HypoE-N1 cells were plated one day before and then exposed to 1 mg/ml FITC-dextran for 2.5 h at 37 °C and 4 °C (as negative control). Scale bars = 50 µm. (**e**) Flow cytometric analysis of FITC-dextran uptake in HypoE-N1 cells. HypoE-N1 cells incubated with FITC-dextran at 37 °C showed significant shift to the right quadrant. (**f**) The right-most open pink histogram represent HypoE-N1 cells incubated with FITC dextran at 37 °C , green histogram indicates HypoE-N1 cells incubated with FITC dextran at 4 °C and the filled purple histogram represent HypoE-N1 cells only at 37 °C. (**g**) Representative image for phagocytosis of apoptotic cell by HypoE-N1 cells during phagocytosis assay after 5 h co-incubation of UV-induced apoptosis RAS^V12^ cells with HypoE-N1 cells at 10:1 ratio. Scale bar = 20 µm. (**h**) Confocal z-stack image (orthogonal projection) for internalization of UV-induced apoptosis RAS^V12^ cells in HypoE-N1 cells. Scale bar = 5 µm. Data are shown as mean ± SEM from two independent experiments (Student’s t-test *p < 0.05).

The phagocytic elimination of cells undergoing apoptosis is a conserved mechanism among multicellular organisms. Defects in phagocytosis processes can lead to structural and functional errors in morphogenesis and tissue homeostasis^35^. We therefore sought to identify the removal of apoptosis-undergoing RAS^V12^ cells in the competitive co-culture. In competitive co-culture, we found RAS^V12^ cells had been engulfed by normal cells (Fig. 6a). To confirm that RAS^V12^ cells were phagocytized by normal cells, we labeled the phagosome with CD68 antibody. We found RAS^V12^ cells were co-localized with CD68 in normal cells which suggested active phagocytosis (Fig. 6b). Next, we quantified phagocytosis by counting co-localization of RAS^V12^ cells with CD68, and our results showed phagocytosis was being promoted in competitive co-culture (Fig. 6c). To demonstrate that HypoE-N1 cells possess phagocytic capacity, we used a FITC-dextran assay and a phagocytosis assay of cells induced to undergo apoptosis by ultraviolet (UV) exposure. In the cells incubated with FITC-dextran, we observed uptake of FITC-dextran into the cells by positive green signals (Fig. 6d). Flow-cytometric analysis of cells incubated with FITC-dextran at 37 °C showed positive signal (Fig. 6e,f). In the phagocytosis assay, cultures of HypoE-N1 cells were stained with red-conjugated phalloidin, a cytoskeletal marker, before co-incubating with UV-induced apoptotic RAS^V12^ cells. Confocal microscopy showed cytoplasmic localization of RAS^V12^ in normal HypoE-N1 cells (Fig. 6g). Analyses of z-stack images revealed that RAS^V12^ cells were phagocytized by normal HypoE-N1 cells, and orthogonal projection view confirmed that RAS^V12^ apoptotic cells were internalized (Fig. 6h). These observations indicate HypoE-N1 cells have phagocytic capacity, and they can remove apoptotic RAS^V12^ cells from competitive co-culture through phagocytosis.

## Discussion

Cell competition is an emerging field for developmental biology. The mechanistic relevance of cell competition addresses unresolved questions during development, for example, organ size and quality control mechanisms to eliminate unfit cells^36^. Despite increasing numbers of cell competition models for development in different organs, there had not been a valid model for cell competition in developing brain. Findings of endogenous cell competition occurring in vivo during epiblast formation^1,37^ had prompted us to investigate the role of cell competition in early brain development. Previous studies demonstrated cell competition in cell lines grown under routine culture conditions, and these have been used as in vitro models with different cell strains including human osteosarcoma U2OS^14^, canine epithelial MDCK^20^ and embryonic fibroblasts NIH3T3^38^. By utilizing HypoE-N1, a neural progenitor cells (NPCs) line, we established an in vitro model system for cell competition in the brain and explored the molecular mechanisms for loser cell elimination.

During brain development, mitotic events and environmental insults lead to somatic mutations which can produce suboptimal cells that can compromise tissue integrity. Competitive interactions between cells have clear benefits in a multicellular organism^39^. RAS oncogene is activated in 30% of human cancers^40^, and H-RasV12 was discovered to be activated in glioblastoma multiforme in adults^41^. We proposed that cell competition act as a quality control mechanism in the developing brain to ensure proper tissue growth and homeostasis maintenance. Studies have reported the potential role of cell competition to eliminate RasV12-transformed cells from epithelia through cell death or apical extrusion^20,42^. RasV12-transformed cells in epithelia trigger non-cell autonomous changes in both types of cells^43^. In this study, we found RAS^V12^ cells demonstrated loss of cellular juvenescence which resulted in the important selective elimination of RAS^V12^ cells through a non-cell autonomous manner. This mechanism of cell competition maintains tissue integrity and acts as quality control by ensuring that cells with deleterious mutations such as RAS^V12^ are eliminated from neuroepithelia during brain development.

Our results showed that introduction of heterogeneity by induction of RAS^V12^ in a small proportion of the cells caused cell competition. Previous studies reported that elimination of loser cells during cell competition was through apoptosis^14,17,19^ with the removal of apoptotic loser cells by phagocytosis^17^. These reports supported our data, which showed that in competitive condition, RAS^V12^ cells were recognized by the surrounding normal cells and eliminated by non-cell autonomous induction of apoptosis followed by phagocytosis by the neighbouring winner cells. Neural progenitor cells are known to be phagocytes, and their phagocytic activity can regulate adult neurogenesis^44^.

We showed in this study that the elimination of RAS^V12^ cells was context-dependent. This was evidenced with our finding in non-competitive condition of RAS^V12^ to RAS^V12^ (CMTPX) co-culture that apoptosis was not observed. Furthermore, we demonstrated neuroepithelial cell competition requires cell–cell contact and was not mediated by soluble factors. Thus, the apoptosis observed in RAS^V12^ cells was the result of cell competition in a non-cell autonomous manner. Currently, the molecular mechanism underlying loser cells elimination is unresolved. In *Drosophila*, expression of *Flower* gene can distinguish loser cells from winner cells by alternative splicing of the FweLose-A/B isoforms in loser cells. This isoform is necessary and sufficient for competition-driven cell elimination^15^. Myc-driven cell competition in mouse epiblast demonstrated BMP signalling was decreased in loser cells^1^. Our study revealed for the first time that during neuroepithelial cell competition, acute loss of Srsf7 and Ezh2 in loser cells preceded their elimination. We hypothesized that the presence of senescent cells in the early developing brain was related to apoptosis, which has been widely reported. Our findings provide evidence that during early brain development the presence of senescent cells losing cellular juvenescence might activate cell competition resulting in cellular discrimination and removal from the neuroepithelia by apoptosis.

Proteasome regulation of the SR protein family is less understood. We hypothesized that the acute loss of Srsf7 in competitive condition observed in this study was linked to ubiquitylation and proteasome degradation. Our results suggested that Srsf7 underwent degradation via the ubiquitin-proteasome pathway. Besides the known function of Srsf7 as a splicing factor, SR proteins have also been identified as regulators of gene expression^33^. Through functional analyses of *Srsf7*, we demonstrated that it was indispensable for cell growth and survival. *Srsf7* suppression induced loss of cellular juvenescence through *Ezh2* suppression at both mRNA and protein levels. The loss of cellular juvenescence was further evidenced with enhancement of senescence-associated proteins P16 and gamma H2AX. Ezh2 suppression has been reported to rapidly elicit DNA damage and trigger the onset of senescence without loss of H3K27me3^45^. Akizu et al. reported that Ezh2 is required for neural progenitor proliferation and has been linked to a number of human diseases including some neurodegenerative disease^46^.

In summary, we have uncovered how cell competition as a quality control mechanism might maintain brain homeostasis. Mechanistically, cellular juvenescence in unwanted loser cells is deregulated during brain development. Further studies should determine whether this molecular mechanism was conserved in other mammalian tissues. The potential of Srsf7 and other JAGs to be drug targets should also be investigated. Cell competition could be exploited to conceive novel therapeutic interventions in neurological diseases that may boost elimination of unwanted and damaged cells.

## Methods

### Antibodies and materials

The following antibodies were used in this study; Srsf7 (Thermo Fisher Scientific, PA5-39482), Ezh2 (Cell Signaling Technology, #5246S), P16 ARC (Abcam, ab51243), γH2AX (Cell Signaling Technology, #9718), RAS (Cell Signaling,#3965S), HA (Abcam, Ab9110), Gapdh (Cell Signaling Technology, #5174), alpha/beta tubulin (Cell signaling Technology, #2148S), Rabbit IgG (Cell Signaling, #3900), active caspase 3 (Abcam, Ab2302), cleaved PARP (Cell Signaling Technology, #9544), CD68 (abcam,ab955), cytochrome-*C* (Santa-cruz,sc-13156), cleaved caspase 3 (Cell Signaling, #9661S) and phospho-p38 (Cell Signaling, #4511S). Phalloidin conjugated-TRITC (Sigma-Aldrich, P1951) was used at 50 µg/ml. Celltracker Green CMFDA dye and Red CMTPX dye (Life Technologies) were used at 10 µM to label the cells. Mitotracker CMXRos (Thermofisher) was used at 100 nM.

### Generation of inducible RAS^V12^-EGFP in HypoE-N1 cells

HypoE-N1 (Cedarlane, #CLU101), a neural progenitor cell line (NPCs) derived from mouse hypothalamus was used in this study. HypoE-N1 cells were cultured in Dulbecco’s modified Eagle’s medium containing 10% fetal bovine serum. HypoE-N1 cells that express doxycycline-inducible RAS^V12^-EGFP were established by serial transfection of pcDNA6/TR and pcDNA4/TO-RAS^V12^-EGFP. The plasmids were gifts from Shunsuke Kon and Yasuyuki Fujita (Hokkaido University, Japan). To induce RAS^V12^ expression, 20 µg/ml of doxycycline (LKT laboratories, Inc) was used.

### Conditioned medium assay

Conditioned media (CM) from HypoE-N1 cells and competitive co-cultures were centrifuged at 200 g for 3 min. Supernatants of CM were collected and then added to pre-cultured RAS^V12^ cells for 24 h. The RAS^V12^ cells had been pre-treated overnight with doxycycline before plating.

### Indirect co-culture assay

ThinCert cell culture membrane inserts for 12-well plates (Greiner bio-one, 665641) were used to keep HypoE-N1 cells and RAS^V12^ cells separated by a membrane filter. 5 × 10^5^ cells/ml per well were seeded in the lower plates (RAS^V12^ cells), and 2 × 10^5^ cells/0.5 ml per well were seeded in the upper transwell insert (HypoE-N1 cells). Cells were pre-cultured overnight and then treated with doxycycline for 24 h.

### Establishment of neuroepithelial cell competition model

For competitive co-culture, normal and RAS^V12^ were co-cultured at 100:1 ratio while for non-competitive condition, RAS^V12^ and RAS^V12^ labeled with CMTPX (red dye) were co-cultured at 100:1 ratio. Both co-culture conditions were established at > 95% confluency and incubated overnight before doxycycline treatment to induce the expression of RAS^V12^-EGFP. We optimized the cell numbers plated in a 6-well plate to create a condition where RAS^V12^ cells were surrounded by normal cells (competitive condition), and RAS^V12^ (CMTPX) cells were surrounded by RAS^V12^ cells (non-competitive condition).

### RNAi-mediated knockdown and overexpression

In knockdown experiments, Lipofectamine RNAiMAX (Invitrogen) was used to transfect the siRNA duplexes at 50 nM: mouse *Srsf7* siRNA1 (Bioneer, AccuTarget siRNA ID142429), siRNA2 (Sigma-Aldrich, siRNA ID SASI_Mm01_00138329.ID1424259), mouse *Ezh2* (Bioneer, AccuTarget siRNA ID 1356125) and siRNA negative control (Applied Biosystems, AM4611). After 48 h, qPCR or western blot analysis were carried out to assess knockdown efficiency. For overexpression experiments, Lipofectamine 2000 (Invitrogen) was used according to the manufacturer’s instruction to transfect plasmid pcDNA-TO-HA-*Srsf7* or control pcDNA3.1-TdTomato into HypoE-N1 cells. The images of live cells were captured using an EVOS FL microscope (Thermo Fisher Scientific). For the purpose of cell growth analysis, numbers of cells per field were counted manually.

### Immunofluorescence staining

Co-culture cells were plated on glass cover slips. The mixture of cells was incubated overnight, followed by doxycycline treatment for 24–72 h. Cells were fixed in 4% paraformaldehyde (PFA) for 10 min at RT, permeabilized with 0.1% Triton X-100 for 2 min at RT, blocked with 2% FBS for 1 h, and incubated with primary antibodies at 4 °C overnight. After three 10-min washes in PBS, cells were incubated with secondary antibodies (anti-rabbit-IgG conjugated to Alexa-546 [Invitrogen, AQ17) or anti-mouse-IgG conjugated to Alexa-647 (Invitrogen, A21235)] at 1:1,000 for 1 h at RT. After three 10-min washes in PBS, coverslips with cells were mounted onto Prolong Gold DAPI (Invitrogen, P36971) on a glass slide. Images were captured by Olympus FV1000 or Leica, TCS SP8 confocal microscopes. ImageJ software was used for image analysis. Positive and negative cells were defined based on fluorescence intensity. Signals in negative controls were set as threshold and cells with fluorescence intensity higher than threshold were considered positive.

### SYTOX blue

Cells were seeded in 6-well plates and induced with 20 µg/ml of doxycycline for 24 h. Culture media were then removed and replaced with media containing 5 nM SYTOX blue. Cells were incubated with SYTOX blue at 37 °C for 30 min in 5% CO_2_ incubator. After incubation, the SYTOX Blue solution was removed and cell images captured using an EVOS fluorescence microscope**.** Numbers of SYTOX blue positive cells were counted in 10 areas and averaged.

### Apoptosis assay

Dead cells that had undergone apoptosis were analyzed after 24 h doxycycline induction by immunofluorescence staining for active caspase-3 and cleaved PARP. Positive cells were counted on five independent 40X fields for each sample and averaged.

### Time-lapse microscopy

Cells were co-cultured on glass-bottom culture dishes (Mat-Tek Corporation) and allowed to attach for 16 h before addition of doxycycline. After 8 h of doxycycline induction, cells were recorded at 10 min/frame interval for 24 h using an Olympus IX83 microscope. Cells were maintained at 37 °C, 5% CO_2_ and 100% humidity during recording. Videos were analyzed by an Olympus CellSens software and processed with Adobe Photoshop and Image J.

### FITC dextran uptake assay

HypoE-N1 cells were incubated with FITC-conjugated dextran (1 mg/ml) (Tokyo Chemical Industry) at 37 °C, or at 4 °C (negative control) for 2 h in culture medium. After washing with PBS supplemented with 1% BSA, cells were prepared for flow cytometric analysis by centrifugation at 300–400 g for 5 min (RT). Cell pellets were then collected and resuspended in PBS/BSA, followed by centrifugation at 300–400 g for 5 min (RT). Pellets were collected and diluted with PBS/BSA to 1 × 10^7^ cells/ml or greater. The diluted cells were transferred into FACs tubes and analyzed by flow cytometry (FACSCalibur, BD Biosciences). HypoE-N1 cells positive for FITC fluorescent signal were considered as cells that had engulfed the dextran.

### Phagocytosis assay

The phagocytosis assay was modified from Lu et al.^44^. Doxycycline (20 ug/ml) was added to HypoE-N1-RAS^V12^-EGFP cell lines for 24 h to induce expression of EGFP-RAS^V12^. RAS^V12^ cells were then dissociated and exposed to ultraviolet light for 15 min to induce apoptosis before incubating with pre-plated HypoE-N1 cells for 4 h. Cells were then washed with cold PBS to stop phagocytosis, fixed in 4% PFA for 10 min at RT, and stained with 50 µg/ml phalloidin-conjugated TRITC in PBS (Sigma-Aldrich, P1951, 30 min at RT) to identify actin. Nuclei were counterstained with Prolong Gold DAPI (Invitrogen, P36971) mounting agent. Images were visualized with an Olympus FV1000 laser confocal microscope under 100 × magnification and Z-stack images were generated with z-step size at 0.5 µm.

### Quantitative RT-PCR

Total RNA was extracted with TRIzol reagent (Thermo Fisher Scientific). Extracted RNA was quantified using a NanoDrop Lite Spectrophotometer (Thermo Fisher Scientific), and reverse transcribed with high-capacity RNA-to-cDNA kit (Applied Biosystems) using a PCR thermal cycler Dice (Takara) according to the manufacturer’s instructions. Synthesized cDNAs were amplified by qPCR using a LightCycler 480 SYBR Green I Master Kit on a LightCycler 480 instrument (Roche). Quality and specificity of the qPCR amplification was determined by a melting curve analysis. Normalization was made to mouse *Tubb5* or *Polr2a*. Primers used for the qPCR are listed in Supplementary Table S1.

### In vivo ubiquitylation assay

To analyze Srsf7 ubiquitylation, HypoE-N1 cells were transfected with a HA-Ubiquitin expressing plasmid, kindly gifted by Toshifumi Morimura (Shiga University of Medical Science). Transfected cells were treated with 5 µM MG132 (Cell Signaling, #2194) for 8 h before cell harvest to enhance ubiquitylation signals. For immunoprecipitation, Dynabeads Protein A (Invitrogen) were incubated with 2 µg Srsf7 and rabbit IgG antibody for 1 h at RT, and then cell lysates were added to the antibody-bead complexes for a further 16 h at 4 °C. Beads were washed with lysis buffer three times followed by elution with 1X Laemmli sample buffer and denaturation at 70 °C for 10 min. The samples were separated in an 8% SDS-PAGE gel followed by western blotting with HA and Srsf7 antibodies.

### Western blot

Proteins were extracted in RIPA lysis buffer (25 mM Tris-HCl pH 7.6, 150 mM NaCl, 1% NP-40, 1% sodium deoxycholate and 0.1% SDS). SDS-polyacrylamide gel electrophoresis (PAGE) was carried out by dissolving lysates in 5 × Laemmli sample buffer, boiling at 95 °C for 2 min, separation of protein samples (20 μg per lane) through 4–15% PAGE gels and transferring separated proteins to Hybond-P PVDF membrane (GE Healthcare). The membranes were incubated with primary antibodies at 4 °C (O/N), followed by appropriate HRP conjugated anti-immunoglobulin antibodies (1 h/RT) (Thermo Fisher Scientific, anti-mouse 32,430 and anti-rabbit 32,460 at 1:1000). The immunoreactive bands were detected using Chemi-Lumi One L, Chemi-Lumi One Super or Chemi-Lumi One Ultra (Nacalai Tesque). Protein bands were visualized using Image Quant LAS 4000 digital imaging system. Uncropped membranes are illustrated in Supplementary Figure S4.

### Statistical analyses

Statistical analyses were performed with Prism 5 statistical software (Graphpad) by using unpaired two-tailed Student’s *t*-tests. The mean ± SEM is presented for all quantified data and *p* values less than 0.05 were considered significant.

## Supplementary information

Supplementary Information.

Supplementary Video.
