# Supplementary material for: Neuroepithelial cell competition triggers loss of cellular juvenescence
Source: Sci Rep. 2020 Oct 22;10:18044. doi: 10.1038/s41598-020-74874-4 (PMC7582913; doi:10.1038/s41598-020-74874-4)
Supplement: Supplementary file 1 — Supplementary Information. [file 41598_2020_74874_MOESM1_ESM.pdf]

Supplementary Information

Neuroepithelial cell competition triggers loss of cellular juvenescence.

Authors

Faidruz Azura Jam[1#], Takao Morimune[1,2#], Atsushi Tsukamura[1,2#], Ayami Tano[1], Yuya Tanaka[1], Yasuhiro Mori[3], Takefumi Yamamoto[3], Masaki Nishimura[1], Ikuo Tooyama[1], Masaki Mori[1*]

#, These authors contributed to this work equally.

Affiliations

[1]Molecular Neuroscience Research Center (MNRC), [2]Department of Pediatrics, [3]Central Research Laboratory, Shiga University of Medical Science, Seta Tsukinowa-cho, Otsu, Shiga, 520-2192, Japan.

Supplementary Figures

a Conditioned medium assay

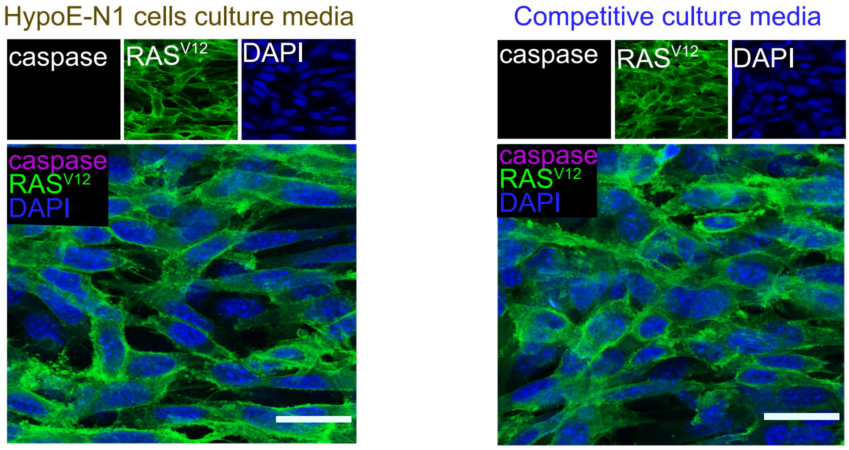

b Indirect co-culture assay (Transwell chambers)

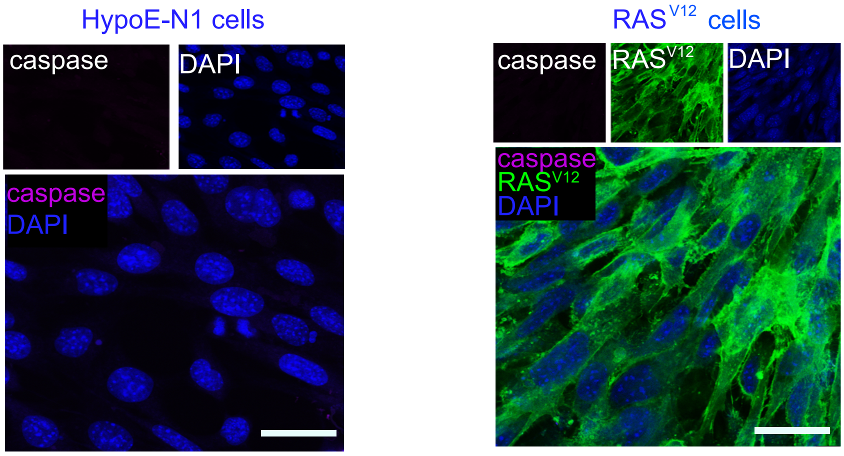

Figure S1. Soluble factors are not involved in neuroepithelial cell competition.
(a) Immunofluorescence images of caspase-3 in RAS^{V12} cells with conditioned media of HypoE-N1 cells culture and competitive cells culture. Scale bars= 30 μm. **(b)** Immunofluorescence images of caspase-3 in HypoE-N1 cells and RAS^{V12} cells in indirect co-culture assay. Scale bars= 30 μm.

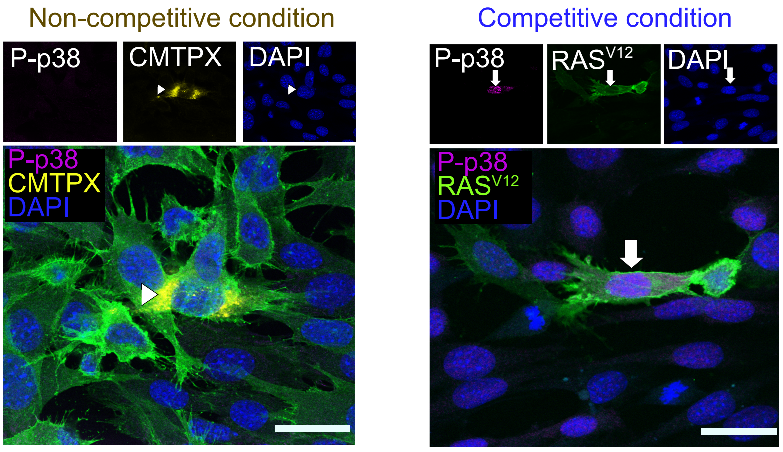

Figure S2. p38 is activated in RAS^{V12} cell during competitive condition. Immunofluorescence images of phospho-p38 in RAS^{V12} cells in non-competitive and competitive condition. Arrow indicate positive for phospho-p38. Scale bars= 30 μm.

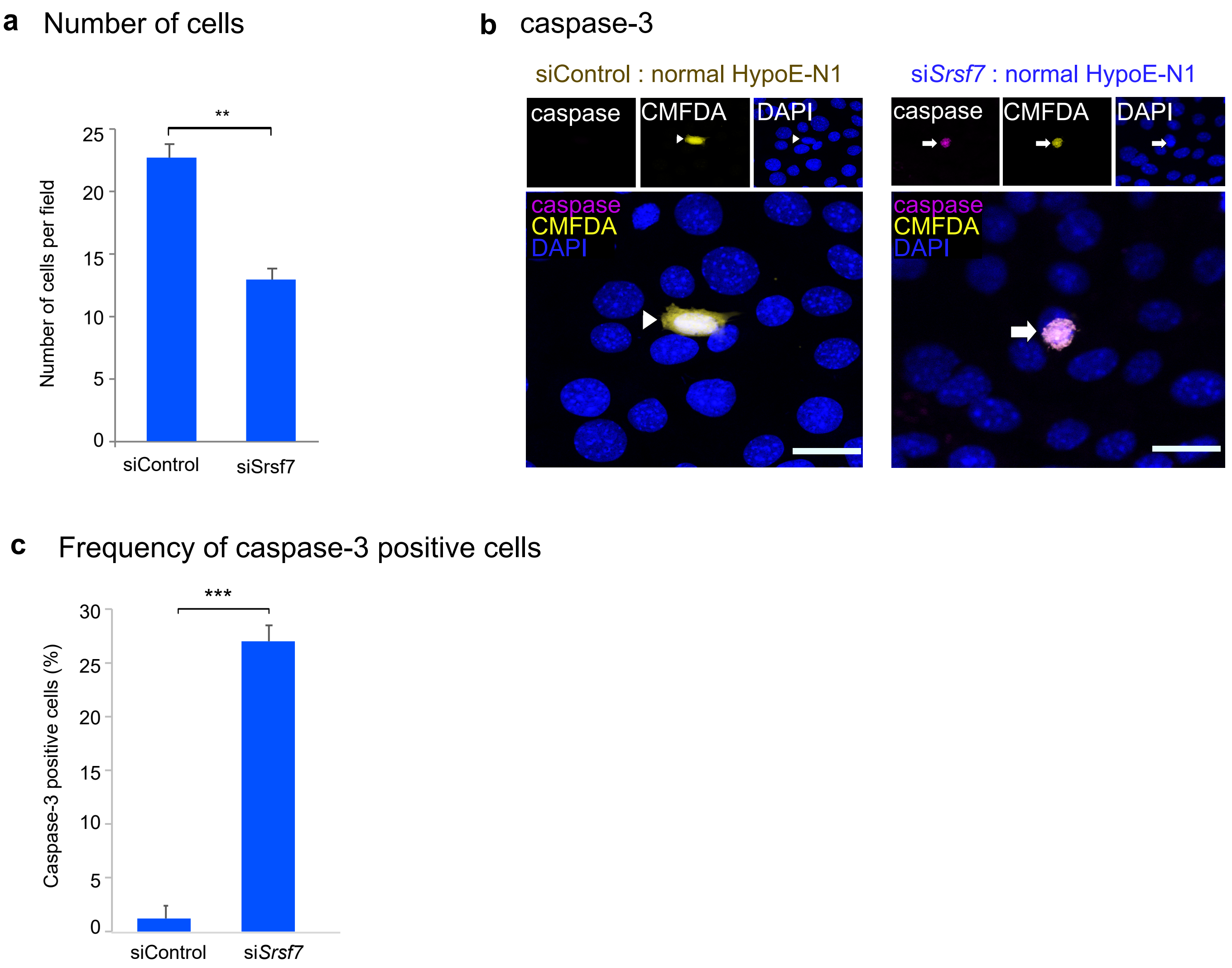

Figure S3. Srsf7 suppression is sufficient to induce cell competition.
(a) Number of HypoE-N1 cells transfected with siControl or si*Srsf7* after 24 h co-cultured with normal cells. **(b)** Representative images of caspase-3 in HypoE-N1 cells transfected with siControl or si*Srsf7* after 24 h co-cultured with normal cells. Arrow indicates positive for caspase-3. Scale bars= 30 μm. **(c)** Frequency of caspase-3 positive in HypoE-N1 cells transfected with siControl or si*Srsf7* after 24 h co-cultured with normal cells. Data are shown as mean ± s.e.m from three independent experiments (Student's t test**p<0.01, ***p<0.001).

Fig. 1b

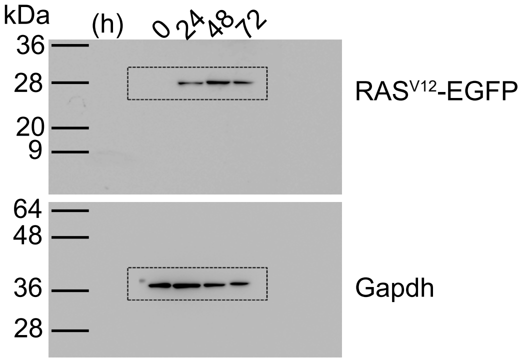

Fig. 3d

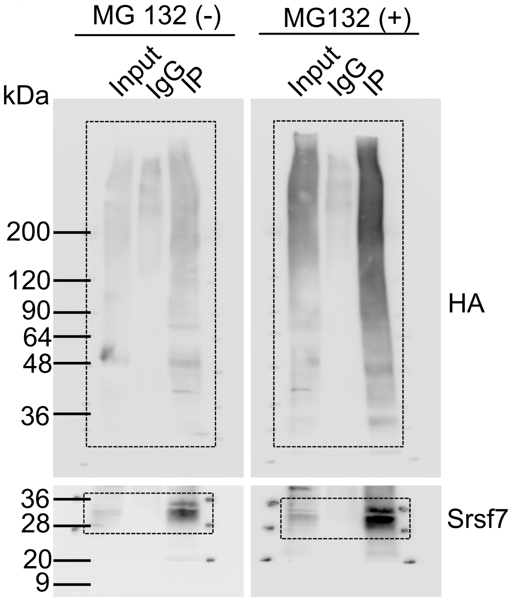

Fig. 5f

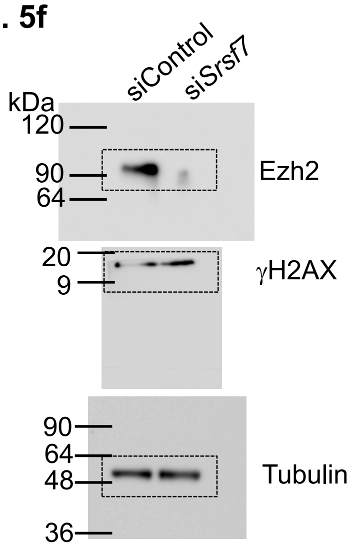

Fig. 3c

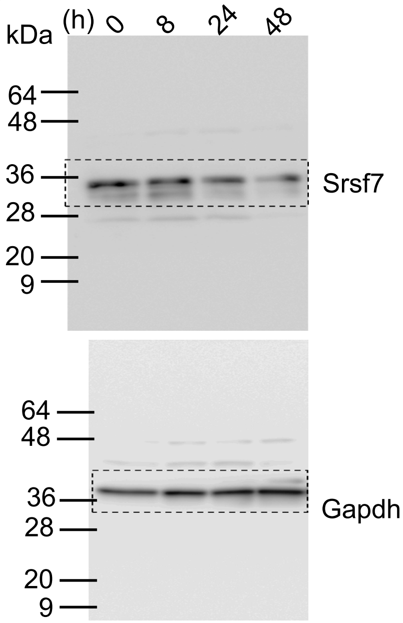

Fig. 4c

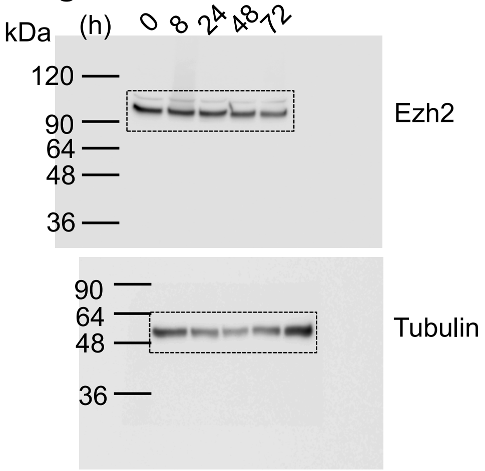

Fig. 5g

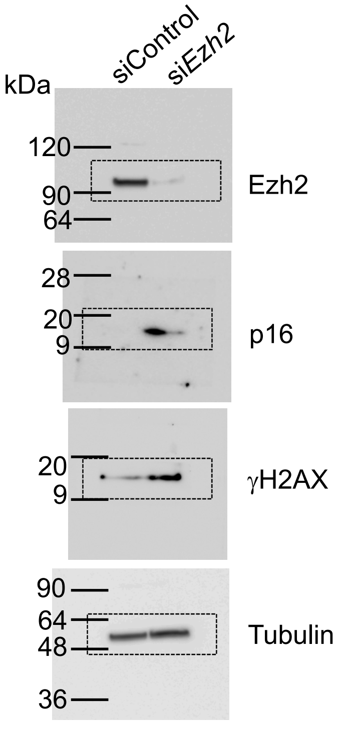

Figure S4. Uncropped western blot membranes. For membranes in figure 5f and 5g , proteins that being probed have different molecular weights. The membranes were cut and probed with different antibodies simultaneously.

Supplementary Table

Gene	Forward	Reverse
Srsf7	5'-ATTCGCCTTTGTGGAATTTG-3'	5'-GGTGGCCTATCAAAACGAGA-3'
Ezh2	5'-CCTGTTCCCACTGAGGATGT-3'	5'-GAGCCGTCCTTTTTCAGTTG-3'
Polr2a	5'-GAGTCCAGAACGAGTGCATGA-3'	5'-ACAGGCAACACTGTGACAATC-3'
Tubb5	5'-GATCGGTGCTAAGTTCTGGGA-3'	5'-AGGGACATACTTGCCACCTGT-3'

Table S1. List of primers for Quantitative RT-PCR.

Supplementary Video

Video S1 (separate file).

Time-lapse video in the competitive co-culture showing elimination of RAS^{V12} cell surrounded by normal cells through cell death induction.
